# Social Determinants of Health: A Need for Evidence-Based Guidelines on How to Capture Data on Underserved Patients

**DOI:** 10.21203/rs.3.rs-2451501/v1

**Published:** 2023-01-12

**Authors:** Nguyen H. Tran, Yahya Almodallal, Mashal Batheja, Nicole Martin, Jennifer Le-Rademacher, Jennifer Ridgeway, Irene G. Sia, Aminah Jatoi

**Affiliations:** Mayo Clinic; Mayo Clinic; Mayo Clinic; Mayo Clinic; Mayo Clinic; Mayo Clinic; Mayo Clinic

**Keywords:** social determinants of health, data capture, Asian, hepatocellular cancer

## Abstract

**Background.:**

Social determinants of health lead to better cancer care. This multi-site, single-institution study sought to capture data on social determinants of health data in Asian Americans with hepatocellular carcinoma; this group constitutes 60% of patients with this malignancy and are often undertreated or not treated at all.

**Methods.:**

This study took advantage of an institutional initiative designed to capture and integrate social determinants of health data into the electronic medical record for all patients. Medical records of Asian Americans with hepatocellular cancer were reviewed to acquire data on housing instability, lack of transportation, financial concerns, and social isolation; a score of 1 indicated poor social determinants of health.

**Results.:**

Of 112 adult Asian American patients with hepatocellular cancer, 22 (20%) were Southeast Asian, and 74 (67%) described English proficiency. A score of 1 (highest risk) was observed in 1 patient (0.9%) for housing instability; 1 (0.9%) lack of transportation; no patient for financial hardship; and 1 (0.9%) for social isolation. However – and importantly – total noncompletion per domain (no question answered within that domain) was observed in 90 patients (80%) for housing instability; 90 (80%) for lack of transportation; 92 (82%) for financial hardship; and 90 (80%) for social isolation. Of note, institution-wide benchmark total noncompletion rates were 0.3%, 0.3%, 47%, and 39% for these respective domains.

**Conclusion.:**

High total noncompletion rates make social determinants of health data challenging to interpret and underscore the need for evidence-based guidelines on how best to capture such data in underserved patients.

## Introduction

Social determinants of health are the social and economic factors that contribute to or detract from the receipt of cancer care [1]. Interrogating a registry of 2392 early-stage cancer patients, Murthy and others examined the likelihood of undergoing potentially curative surgery for early-stage liver cancer [2]. These investigators reported that only 36% of patients underwent surgery and that social determinants of health – specifically, having insurance and stable housing – predicted whether patients underwent a potentially curative surgical procedure. Such findings point to the importance of capturing information on social determinants of health, as these factors are pivotal to enabling patients to receive much-needed cancer care.

Although studies are only starting to emerge among Asian Americans, social determinants of health might be of particular importance to these underserved patients. Trinh and others examined rates of cancer screening in several million individuals and reported that, despite higher education levels and higher income levels, Asian Americans were less likely to undergo screening for colorectal (odd ratio (OR): 0.78 (95% confidence interval (CI): 0.63, 0.96)), cervical (OR:0.45 (95% CI: 0.36, 0.55)), and prostate cancer (OR: 0.55 (95% CI: 0.39, 0.78)), presumably and in part because of “limitations in healthcare access” [3]. Despite this study’s robust sample size and despite the salient nature of these findings, few studies have sought to examine social determinants of health in Asian American patients, who have already been diagnosed with cancer, an observation that suggests a need to focus on this topic in these patients.

The current study specifically focused on Asian American patients with hepatocellular cancer because these patients are at high risk for this malignancy and, in fact, constitute 60% of patients with this cancer in the United States [4, 5]. Further, Asian American patients are often diagnosed with late-stage hepatocellular cancer, and the majority are either undertreated or not treated at all [6, 7]. These untoward circumstances – coupled with a paucity of data on social determinants of health in this population – prompted this current study.

## Methods

### Overview.

The Mayo Clinic Institutional Review Board approved this single-institution, multi-site study (#21-002052), which encompassed healthcare sites in Minnesota, Arizona, Florida, Iowa, and Wisconsin. A unified electronic medical record remains integral to medical care at all these sites.

### Medical Record Ascertainment and Data Acquisition.

A previously constructed, comprehensive registry that included consecutive Asian American patients with hepatocellular carcinoma was used to identify patients. This registry focused only on Asian American adult patients who had been diagnosed with hepatocellular carcinoma between January 2011 and December 2020 with follow-up through 2021; by design, the registry included extensive data on ethnicity and English language proficiency.

Because an institutional initiative to gather systematic information on social determinants of health was implemented in June of 2019, the current study focused on patients who received medical care or follow up between 2019 and 2021. The medical records of all these patients were then rereviewed in 2022 with the express purpose of reporting on social determinants of health.

Data acquisition on the domains of housing instability, lack of transportation, financial concerns, and social isolation were acquired from the medical record. These domains were chosen because they are germane to patients with hepatocellular cancer and to the acquisition of cancer care in general cancer populations, as described previously [8].

### Patient Questionnaire Completion.

All patients had been asked to complete a questionnaire on social determinants of health as part of their clinical care. The questionnaire was administered via an electronic tablet to patients once per year immediately prior to a clinic visit. With the COVID-19 pandemic, virtual completion was implemented for those patients who underwent virtual visits. Virtual completion entailed sending the questionnaire to patients via a secure electronic portal that enabled the patient to access his or her own medical record; an email or text message reminder was sent to ask the patient to complete the questionnaire prior to the clinic visit.

### Social Determinants of Health Questions.

The questionnaire was developed in English as a multiinstitutional initiative. The questionnaire initially included 11 domains with others added later. In total, it included approximately 50 questions with others added later. Again, the current study focused only on the 4 domains mentioned above.

The questionnaire included several items that pertained to each of these 4 domains (Supplementary Table 1). For example, “In the past 12 months, was there a time when you were not able to pay the mortgage or rent on time?,” was used to help assess housing instability; “In the past 12 months, has lack of transportation kept you from medical appointments or from getting medications?,” was used to help assess lack of transportation; “How hard is it for you to pay for the very basics like food, housing, medical care, and heating,” was used to help assess financial concerns; and “How often do you get together with friends or relatives?,” was used to help assess social isolation. Each of these questions was accompanied by check boxes for the patient to complete.

### Summary of Social Determinants of Health Data.

Within the electronic medical record, each domain for each patient was summarized with a single score derived by weighted answers to each question relevant to that domain. This score ranged from 1–2 for housing instability and for lack of transportation and from 1–3 for financial concerns and for social isolation. Lower scores were of greater concern with a score of 1 indicative of high risk for poor social determinants of health.

For the current study, completion of questions per domain was categorized as total non-completion when no question within a domain was answered and as partial completion when only some questions within a domain were answered. Rates of total noncompletion per domain are derived from the number of patients with total noncompletion per the total number of Asian American patients with hepatocellular cancer. Similarly, rates of partial noncompletion are derived from the number of patients with partial noncompletion per the total number of Asian American patients with hepatocellular cancer.

### Descriptive Data Presentation.

The primary goal was to provide descriptive data on social determinants of health in Asian American patients with hepatocellular cancer. Formal comparisons with institution-wide benchmark data were neither planned nor undertaken, as benchmark data were provided only as estimates from approximately 2.9 million unique patients who received care within the prior 3 years.

## Results

### Demographics.

A total of 112 patients met the study inclusion criteria and are the focus of this report. The median age at cancer diagnosis was 60 years (range: 18, 79 years) with 80 (71%) men and 32 (29%) women (Table 1). Twenty-two patients (20%) were Southeast Asian, and 74 (67%) described English proficiency (Table 1).

### Social Determinants of Health.

Scores for each of the 4 domains of interest include high risk – or a score of 1 – for housing instability in a single patient (0.9%); lack of transportation in a single patient (0.9%); financial hardship in no patient (0%); and social isolation in 1 patient (0.9%) (Table 2).

Notably, for housing instability, total noncompletion and partial noncompletion occurred in 90 patients (80%) and 2 patients (2%), respectively. For lack of transportation, these same numbers and percentages of total noncompletion and partial noncompletion were observed. Consistently, for financial hardship, total noncompletion and partial noncompletion were seen in 92 (82%) and 2 patients (2%), respectively. For social isolation, total noncompletion and partial noncompletion were seen in 90 (80%) and 4 (4%) of patients, respectively.

### Institutional Benchmark Data.

Benchmark data on the percentage of patients who completed the questionnaires relied on approximately 2.9 million total patients and showed total noncompletion rates of 0.3%, 0.3%, 47%, and 39% for the domains of housing instability, lack of transportation, financial concerns, and social isolation, respectively. Partial noncompletion rates for these domains were reported as 41%, 45%, 3%, and 11%, respectively.

## Discussion

This study found surprisingly sparse data on social determinants of health among Asian American patients with hepatocellular cancer, despite institutional efforts to gather such data in a systemic matter. Although our intention was not to compare the percentage of data capture between Asian American patients and the general patient population, it is sobering to report that, in the general population, questionnaire total noncompletion rates in these domains occurred in less than 50% – and sometimes in less than 1% – whereas in Asian American patients with hepatocellular cancer, these rates were consistently 80% or higher. Although it appears that only a single Asian American patient – or fewer – in each domain was at high risk for one of the 4 domains of interest, this observation provides little reassurance in view of such astoundingly high rates of total noncompletion. Thus, drawing meaningful conclusions on social determinants of health in these Asian American patients with hepatocellular carcinoma is challenging.

We can only speculate on what accounts for these high total noncompletion rates. First, demographic data from the medical record show that 67% of Asian American patients reported English proficiency. However, completing a 50+ item questionnaire might pose challenges for any patient but particularly for those who speak English but not as their first language. The length of the questionnaire might unwittingly have led to disparities in completion rates for those who are proficient in English but nonetheless find such a long questionnaire taxing. Admittedly, a multilingual questionnaire and a shorter set of questions could perhaps have made questionnaire completion easier for patients. Second, patients with hepatocellular cancer are at times highly symptomatic from their disease. Asking patients to complete an extensive questionnaire when they do not feel well might also explain such a high rate of total noncompletion. Third, at times patients were asked to log onto a computer and complete the questionnaire at home. Doing so might have added an extra burden to patients with hepatocellular cancer, particularly, again, when they might not have been feeling well. Fourth, cultural aspects of questionnaire completion might also explain these disappointingly high total noncompletion rates among Asian American patients. In data from the general population, we observed that noncompletion rates were at their highest at 47% on the topic of financial concerns, a finding that seems to coincide with a reluctance at large to talk about personal finances in our society [10]. Such data suggest that cultural issues – perhaps relevant to a reluctance to respond to all these domains - might also be at work in explaining these high noncompletion rates in Asian American patients with hepatocellular cancer.

Furthermore, a certain irony seems to be at play here. Healthcare providers appear to expect that patients with compromised social determinants of health — which interfere with patients’ ability to seek help for themselves — will surmount such extant challenges for the express purpose of completing a social determinants of health questionnaire. Underserved patients who are struggling with social determinants of health are likely also to be struggling with questionnaire completion. An in depth understanding of the obstacles that account for total and partial noncompletion of questionnaire domains could enhance our understanding of issues that are directly pertinent to the health of these patients. Indeed, the high rates of total noncompletion reported here call for further investigation, potentially with the use of qualitative research methods, and, ultimately, with the goal of developing guidelines to instruct how best to capture such important information from underserved patients. Understanding the challenges inherent in data acquisition could be key to helping Asian American patients with hepatocellular cancer receive better cancer care and could perhaps also help other underserved patients.

Finally, this study has limitations that include a relatively small sample size, a single institution setting, the acquisition of data from a tertiary medical center setting, and a comparative group that is comprised of general population of patients (as opposed to another subgroup of Asian patients). These limitations might be interfering with the generalizability of findings, but they also raise the possibility that such total noncompletion rates could be even more severe and pervasive in other medical centers. Undoubtedly, social determinants of health merit further study in Asian American patients with hepatocellular cancer with the goal of understanding how best to capture these data and, ultimately, how best to help these underserved patients and others.

## Figures and Tables

**Table 1:** Patient Demographics (n = 112)

CHARACTERISTIC	
Median age (range) at cancer diagnosis**	61 (20, 87)
Sex	80 (71)
male	32 (29)
female	
Vital status at medical record review	93 (83)
alive at final medical record review	19 (17)
deceased	
Ethnicity	17 (20)
Chinese	8 (9)
Vietnamese	7 (8)
Cambodian	4 (5)
Laotian	2 (2)
Thai	1 (1)
Hmong	47 (55)
Other/not provided in medical record	
Language proficiency	74 (68)
English	8 (7)
Chinese	6 (6)
Cambodian	4 (4)
Vietnamese	4 (4)
Laotian	2 (2)
Hmong	1 (1)
Thai	10 (9)
Other	

**Table 2:** Social Determinants of Health Scores and Patient Completion

DOMAIN	PATIENT SCORES (%)		NUMBER OF PATIENTS WITH TOTAL NONCOMPLETION (%)	NUMBER OF PATIENTS WITH PARTIAL NONCOMPLETION (%)	INSTITUTIONAL BENCHMARK** DATA ON TOTAL NONCOMPLETION %	INSTITUTIONAL BENCHMARK** DATA ON PARTIAL NONCOMPLETION %
Housing Instability	1*	1 (0.8)	90 (80)	2 (2)	0.3	41
	2	19 (16)				
Lack of Transportation	1	1 (0.8)	90 (80)	2 (2)	0.3	45
	2	19 (17)				
Financial Concerns	1	0	92 (82)	2 (2)	47	3
	2	3 (3)				
	3	15 (13)				
Social Isolation	1	1 (0.8)	90 (80)	4 (4)	39	11
	2	6 (5)				
	3	11 (10)				

* Lower scores denote higher risk for compromised social determinants of health.

** Benchmark data are based on all of the estimated 2.9 million unique patients served by the institution over the past 3 years.

## References

[1] Dsouza JP, Van den Broucke S, Pattanshetty S, Dhoore W. Factors explaining men's intentions to support their partner's participation in cervical cancer screening. BMC Womens Health. 2022 Nov 11;22(1):443. doi: 10.1186/s12905-022-02019-y. 36369003 PMC9652784

[2] Murthy SS, Ortiz A, DuBois T, Sorice KA, Nguyen M, Castellanos JA, Pinheiro P, Gonzalez ET, Lynch SM. The effect of social determinants of health on utilization of surgical treatment for hepatocellular carcinoma patients. Am J Surg. 2022 Oct 12:S0002-9610(22)00641-9. doi: 10.1016/j.amjsurg.2022.10.011. Epub ahead of print. 36344305

[3] Trinh QD, Li H, Meyer CP, Hanske J, Choueiri TK, Reznor G, Lipsitz SR, Kibel AS, Han PK, Nguyen PL, Menon M, Sammon JD. Determinants of cancer screening in Asian-Americans. Cancer Causes Control. 2016 Aug;27(8):989–98. doi: 10.1007/s10552-016-0776-8. Epub 2016 Jul 2. 27372292

[4] Cancer and Asian Americans - The Office of Minority Health (hhs.gov); https://minorityhealth.hhs.gov/omh/browse.aspx?lvl=4&lvlID=46#:~:text=Cancer%20Incidence%20Rates%20per%20100%2C000%20%E2%80%93%20Men%20,%20%200.7%20%203%20more%20rows%20; last accessed December 3, 2022.

[5] Medina HN, Callahan KE, Morris CR, Thompson CA, Siweya A, Pinheiro PS. Cancer Mortality Disparities among Asian American and Native Hawaiian/Pacific Islander Populations in California. Cancer Epidemiol Biomarkers Prev. 2021 Jul;30(7):1387–1396. doi: 10.1158/1055-9965.EPI-20-1528. Epub 2021 Apr 20. 33879454 PMC8254771

[6] Torre LA, Sauer AM, Chen MS Jr, Kagawa-Singer M, Jemal A, Siegel RL. Cancer statistics for Asian Americans, Native Hawaiians, and Pacific Islanders, 2016: Converging incidence in males and females. CA Cancer J Clin. 2016 May;66(3):182–202. doi: 10.3322/caac.21335. Epub 2016 Jan 14. 26766789 PMC5325676

[7] Thompson CA, Gomez SL, Hastings KG, Kapphahn K, Yu P, Shariff-Marco S, Bhatt AS, Wakelee HA, Patel MI, Cullen MR, Palaniappan LP. The Burden of Cancer in Asian Americans: A Report of National Mortality Trends by Asian Ethnicity. Cancer Epidemiol Biomarkers Prev. 2016 Oct;25(10):1371–1382. doi: 10.1158/1055-9965.EPI-16-0167. 27694108 PMC5218595

[8] Zettler ME, Feinberg BA, Jeune-Smith Y, Gajra A. Impact of social determinants of health on cancer care: a survey of community oncologists. BMJ Open. 2021 Oct 6;11(10):e049259. doi: 10.1136/bmjopen-2021-049259. PMC8496396 34615676

[9] Pinheiro LC, Reshetnyak E, Akinyemiju T, Phillips E, Safford MM. Social determinants of health and cancer mortality in the Reasons for Geographic and Racial Differences in Stroke (REGARDS) cohort study. Cancer. 2022 Jan 1;128(1):122–130. doi: 10.1002/cncr.33894. Epub 2021 Sep 3. 34478162 PMC9301452

[10] https://www.theatlantic.com/family/archive/2020/03/americans-dont-talk-about-money-taboo/607273/; last accessed December 3, 2022

